# A Narrative Review on Neuro-Ophthalmological Manifestations That May Occur during Pregnancy

**DOI:** 10.3390/life14040431

**Published:** 2024-03-24

**Authors:** Nicoleta Anton, Camelia Margareta Bogdănici, Daniel Constantin Branișteanu, Theodora Armeanu, Ovidiu-Dumitru Ilie, Bogdan Doroftei

**Affiliations:** 1 Ophthalmology Department, Faculty of Medicine, University of Medicine and Pharmacy “Grigore T. Popa”, University Street, No. 16, 700115 Iasi, Romania; dbranisteanu@yahoo.com; 2 Ophthalmology Clinic, Sf. Spiridon Emergency Clinical Hospital of Iaşi, 700111 Iași, Romania; 3 Department of Mother and Child, Faculty of Medicine, University of Medicine and Pharmacy “Grigore T. Popa”, University Street, No. 16, 700115 Iasi, Romania; theodoraarmeanu@yahoo.com (T.A.); ovidiuilie90@yahoo.com (O.-D.I.); bogdandoroftei@gmail.com (B.D.); 4 Origyn Fertility Center, Palace Street, No. 3C, 700032 Iasi, Romania

**Keywords:** pregnancy, papilledema, cranial neuropathies, eclampsia/preeclampsia, pituitary apoplexy

## Abstract

Aim: As a medical condition, pregnancy mandates the simultaneous treatment of both the mother and the fetus, making it a distinctive aspect of clinical medicine. Material and Method: We analyze the physiological changes occurring in the eyes and brain during pregnancy, as well as the neuro-ophthalmological manifestations that can occur during pregnancy. Studies published in both English and other languages, case reports, and reviews from 2011 to 2023 onwards were included. All surveys were acquired by exploring the databases. Results: We found a total of 2135 articles that showcase neuro-ophthalmic changes related to pregnancy: review and research articles (Science Direct 804, Web of Science 923, Scopus 345, and 63 Pub Med). In total, 86 studies were examined after applying the inclusion and exclusion criteria. Bilateral papilledema can be a warning sign for intracranial hypertension or cerebral venous sinus thrombosis. Additionally, when unilateral, it is important to differentially diagnose anterior ischemic optic neuropathy secondary to a hypercoagulant, compressive or inflammatory optic neuropathy, optic neuritis, or even orbital pseudotumor state. Severe eclampsia and preeclampsia can manifest as choroidal infarction, serous retinal detachment, and even cortical blindness. There can also be implications at the level of cranial nerves or transient Horner syndrome. Conclusions: Evaluating and treating a pregnant woman with neuro-ophthalmological manifestations is challenging. The obstetrician closely follows and has a medical relationship with the pregnant woman; hence he/she might be the first to be informed about the general condition of the pregnant woman or might request an ophthalmologic examination tailored to each specific case.

## 1. Introduction

The physiological changes experienced during pregnancy have far-reaching implications for various body systems. Generally, due to the influence of pregnancy hormones, several pre-existing conditions can worsen. Studies show that tumors can increase in size, leading to the emergence of neuro-ophthalmic symptoms. Additionally, autoimmune conditions tend to improve during pregnancy but worsen after childbirth. Supporting a pregnant woman dealing with a neuro-ophthalmic disorder poses unique challenges.

Throughout pregnancy, there is a potential for blood volume and cardiac output to nearly double. Patients undergo a significant weight increase within a short duration, alongside a substantial surge in extracellular fluid as serum osmolality decreases notably. Fibrinolysis-related function or process decreases, while coagulation factors rise, and cellular immunity declines across different body systems during pregnancy. These physiological shifts lead to an increased occurrence of vascular diseases. This includes conditions like retinal artery occlusion, spontaneous orbital hemorrhage, and pituitary apoplexy, among others [1,2,3,4,5,6].

Another explanation for vascular changes during pregnancy is that the preparation for handling blood loss during childbirth involves increased levels of plasminogen, fibrinogen, and factors I, V, VII, IX, and X, alongside a decrease in fibrinolysis. The expansion of the uterus is facilitated by the increase in smooth muscle cells, while the breakdown of reticular fibers within the vascular linings increases the risk of vascular complications.

Immunologically, especially postpartum, issues can deteriorate. For example, women diagnosed with multiple sclerosis are at a greater risk of experiencing a demyelination episode [7].

Many of these changes occur directly in the eye, including alterations in refraction due to hormonal changes in corneal fluids, changes in nearsightedness, affected accommodation, and reduced pressure within the eye due to low episcleral venous pressure and heightened aqueous flow. Additionally, other visual changes may occur as secondary effects, including conditions like meningioma, pituitary adenoma, demyelinating disorders, myasthenia gravis (MG), thyroid conditions, idiopathic intracranial hypertension (IIH), cerebral venous sinus thrombosis (CVST), cranial and vascular conditions, and optic nerve issues, among others.

Regarding neuro-ophthalmological aspects, the most common changes are associated with pregnancy risk factors, pregnancy-induced hypertension, severe preeclampsia, and eclampsia. Eclampsia and preeclampsia involve severe hypertension and proteinuria, typically occurring after the 20th week of gestation. The reported cases involve retinopathy with Purtscher-like changes associated with a serous retinal detachment that occurs in pregnant women recognized with preeclampsia, as well as those secondary to HELLP syndrome [6,7,8].

Optic nerve impairment during pregnancy can manifest as optic neuritis, either independently or as an early sign of systemic demyelinating disorders such as multiple sclerosis (MS) and neuromyelitis optica (NMO). As pregnancy induces significant immunological and hormonal changes crucial for the fetus, immunological conditions like multiple sclerosis and neuromyelitis optica may occur more frequently immediately after childbirth. Nevertheless, specialized literature also discusses rare occurrences of optic neuritis attacks during the first trimester of pregnancy and rare cases of myelin oligodendrocyte glycoprotein optic neuritis (MOG-ON). Identifying AQP4-Ab (aquaporin 4-antigen) and MOG-Ab (oligodendrocyte glycoprotein antibodies) is pivotal for individuals having optic neuritis undergoing various therapies, as these may lead to different prognoses [6,7,8].

Idiopathic intracranial hypertension (IIH) commonly affects females in their reproductive stage, with increased weight being a substantial risk element for the emergence or exacerbation of the condition. Managing weight gain during pregnancy is crucial due to the potential exacerbation of IIH, increased retention of fat postpartum, and the potential for maternal and fetal complications [9,10,11]. A neuro-ophthalmological diagnosis is critical before initiating suitable treatment. Treatment plans should consider the implications for both the woman and the fetus [12]. This review focuses on articles discussing neuro-ophthalmological changes that arise or worsen while pregnant.

## 2. Materials and Methods

This manuscript followed the procedures outlined earlier by Green et al. [13].

### 2.1. Querying Databases

Between 2011 and 2023, all surveys were acquired by exploring the following databases: PubMed/Medline, ISI Web of Knowledge, Science Direct, and Scopus. The search strategy included the following keywords to find suitable materials: “pregnancy and papilledema”, “pregnancy and preeclampsia”, “pregnancy and neuro-ophthalmology”, “eye disease in pregnancy”, the visual system and “preeclampsia and eclampsia”, and “optic nerve disorders and preeclampsia”. The PubMed query used was (pregnancy [Title/Abstract] AND preeclampsia [Title/Abstract]) AND [Title/Abstract] AND neuro-ophthalmology [Title/Abstract] AND papilledema [Title/Abstract] AND eye disease [Title/Abstract] AND optic neuritis [Title/Abstract].

### 2.2. Qualification Standards

Studies published in both English and other languages, case reports, reviews, and animal studies from 2011 to 2023 onwards were included. Exclusions consisted of non-English articles, editorial letters, conference posters, preprints, and computational simulations.

### 2.3. Selection Process

Papers meeting the criteria were initially screened based on their titles and abstracts. Subsequently, these entries underwent a thorough content evaluation, resolving any discrepancies or conflicting viewpoints through joint approval by two expert specialists, N.A. and B.D.

## 3. Results and Discussion

**Figure 1:**
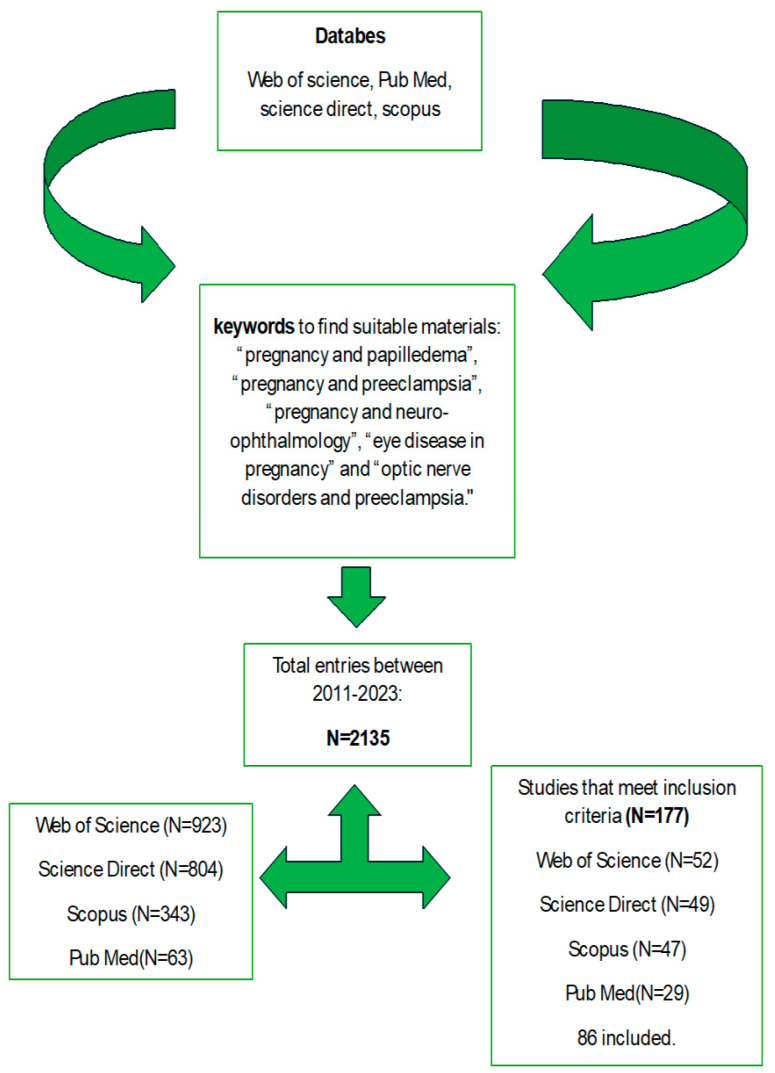
An overview of all studies and entries developed through the combined use of keywords and various searched databases. A total of 86 articles were considered for analysis.

We found a total of 2135 articles that showcase neuro-ophthalmic changes related to pregnancy: review and research articles (Science Direct 804, Web of Science 923, Scopus 345, and 63 Pub Med). In total, 86 studies were examined after applying the inclusion and exclusion criteria. An overview of all studies and entries developed through the combined use of keywords and various searched databases is presented in Figure 1.

### 3.1. Brief Review of Eye Disease in Pregnancy

Pregnancy induces physiological changes across all maternal organ systems, including the eyes. Though most changes are benign, they may cause visual symptoms, leading patients to seek medical evaluation.

In pregnancy, there are observed anatomical and physiological changes within the eyes. Certain pre-existing eye conditions are known to either deteriorate or improve during this period, though these changes are typically temporary. What further complicates diagnosis and management is the limitation that ophthalmic medications commonly prescribed for individuals who are not pregnant cannot be used in the same manner during pregnancy or while breastfeeding due to associated risks to the fetus [6,14,15,16].

The eye changes experienced during pregnancy can be divided into physiological and pathological categories. Pathological changes are further classified into different groups: ophthalmic alterations occurring for the first time during pregnancy (such as keratoconus, dry eye syndrome, etc.), pre-existing eye conditions that are influenced by pregnancy (including glaucoma, diabetic retinopathy, and neuro-ophthalmic issues), and eye manifestations of systemic diseases. These systemic diseases include conditions specifically related to pregnancy, like preeclampsia/eclampsia/Sheehan syndrome, and those more common during pregnancy, such as idiopathic intracranial hypertension and disseminated intravascular coagulation [6,14,15,16].

Physiological changes mainly involve increased pigmentation around the eyes and facial pigmentation during pregnancy, often referred to as the mask of pregnancy, chloasma, or melasma. These changes are a result of heightened levels of estrogen, progesterone, and melanocyte-stimulating hormone. Additionally, unilateral ptosis may occur due to fluid accumulation and hormonal effects on the levator aponeurosis, typically resolving after childbirth. Pregnancy can also impact tear film physiology, potentially leading to the development of dry eye syndrome. This could be associated with increased immune response in lacrimal duct cells and direct damage to acinar cells caused by prolactin, beta-1 growth factor, and epidermal growth factor. Changes in corneal curvature might also occur, reaching their peak in late pregnancy and resolving after childbirth and the breastfeeding period. These corneal curvature changes may induce a myopic shift, temporary loss of accommodation, and reduced amplitude. Consequently, it is advisable to avoid altering prescriptions during pregnancy and breastfeeding for several months and refrain from refractive surgery during this period [14,15,16].

It is intriguing that during pregnancy, intraocular pressure drops by 19.6% among those with typical intraocular pressure and by 24.4% in those diagnosed with high eye pressure. One explanation is the decrease in pressure within the episcleral vessels during pregnancy, which is attributable to reduced scleral rigidity and increased tissue elasticity. In individuals with glaucoma, intraocular pressure tends to improve during pregnancy, although some studies have reported refractory cases. Notably, antiglaucoma medications carry teratogenic risks and are contraindicated for pregnant women. Therefore, laser trabeculoplasty is recommended as a means to manage intraocular pressure before a glaucoma patient plans pregnancy [14,15,16,17,18].

Systematically, pregnancy triggers various immunological changes in the maternal body. Maternal immunity tends to favor a TH2 humoral response, where TH2 cytokines protect the fetus at the maternal–fetal interface. Moreover, regulatory T-cells elevate their activity levels at this interface, which are crucial for maintaining adequate immunosuppression to prevent potential harm to the fetus. Consequently, certain pre-existing ophthalmic conditions may improve during pregnancy, while others might exacerbate due to these immunological changes. Table 1 outlines the influence of pregnancy on pre-existing ophthalmological conditions.

Pregnancy-induced high blood pressure is connected with several clinically relevant alterations. These encompass a range of changes including conjunctival vascular anomalies, hypertensive retinopathy, exudative detachment of the retina, hemorrhage in the vitreous or preretinal space, optic neuropathy due to ischemia, and hypertensive choroidopathy. Some optic nerve alterations are specifically linked to low birth weight and APGAR scores. Studies emphasize the critical role of ophthalmologic assessments in pregnant women with such conditions to prevent secondary fetal effects [6,14,16,23].

Vascular occlusive diseases encompass various conditions like blockage in retinal arteries, blockage in retinal veins, disseminated intravascular coagulation (DIC), thrombotic thrombocytopenic purpura (TTP), antiphospholipid antibody syndrome (APS), amniotic fluid embolism, and thrombosis in cerebral veins. These conditions arise due to the hypercoagulable state during pregnancy. Although rare, central and branch retinal artery occlusions related to pregnancy have been reported. Retinal vein occlusions are much less common than arterial ones [3,23]. Research indicates that retinal artery occlusions are infrequent in individuals under 30 years old. For instance, Yoo-Ri Chung described a scenario that involved a healthy 29-year-old pregnant woman at 12 weeks gestation who presented with reduced vision in her left eye due to an inferotemporal branch retinal artery occlusion and macular edema. Further investigations uncovered a coagulability disorder, indicated by Factor VIII activity at 220% (normal range 60–140%). Within 2 months, her visual acuity improved from finger counting to 20/30, and optical coherence tomography confirmed the resolution of macular edema. This case suggests that branch retinal artery occlusion can manifest in healthy individuals without systemic or ocular disorders, despite extensive evaluations [3].

Other pre-existing conditions accentuated by pregnancy include pituitary adenomas, meningiomas, and uveal melanomas. Asymptomatic or microadenomatous pituitary adenomas can increase in size during pregnancy, leading to symptoms such as headaches, changes in visual fields (bitemporal hemianopsia), reduced visual acuity, and the onset of double vision. In these cases, regular ophthalmologic evaluation of the pregnant woman is recommended [16]. Progesterone and estrogens might mediate the growth and vascularization of certain pre-existing meningiomas during the latter stages of pregnancy. Exacerbation of Graves’ disease may occur during the first trimester [6,14,16].

### 3.2. Neuro-Ophthalmologic Manifestations in Pregnancy

The evaluation of central nervous system disorders does not differ from that of a non-pregnant individual. However, pregnancy can worsen pre-existing conditions, making the management of neuro-ophthalmologic conditions challenging during pregnancy. This challenge arises from the limitations in conducting certain imaging investigations and the constraints in administering several medications, especially during the first trimester.

**Figure 2:**
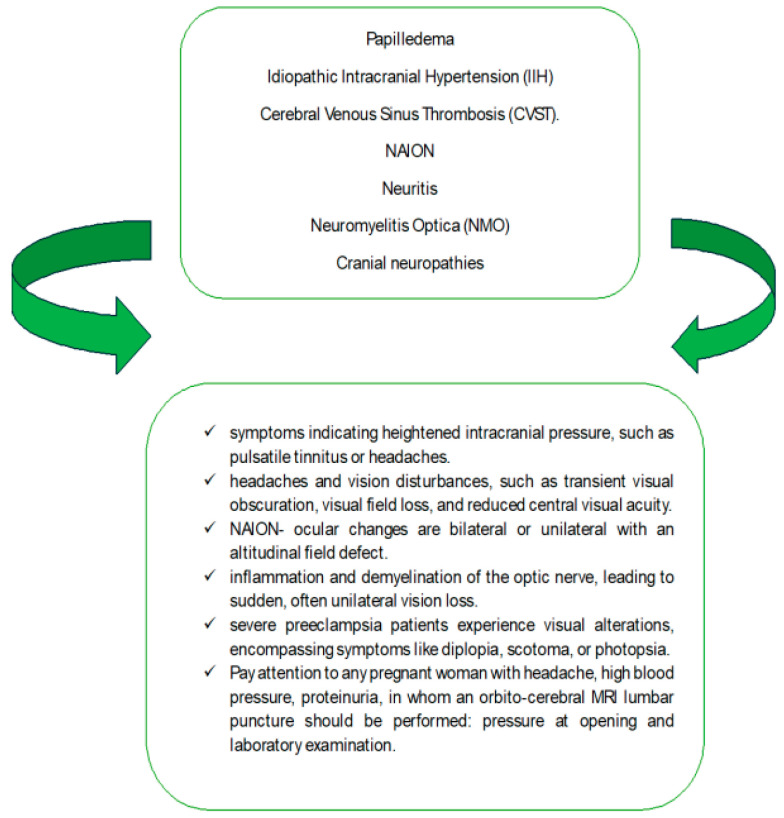
Neuro-ophthalmic symptoms of frequent pathology in pregnant women.

In Figure 2, the neuro-ophthalmic symptoms of frequent pathology in pregnant women are summarized.

#### 3.2.1. Papilledema and Pregnancy

Papilledema refers to swelling of the optic disc due to heightened intracranial pressure. (ICP). It is considered a medical emergency due to its association with severe neurological sequelae and visual acuity loss. The primary causes of this swelling are similar to those in non-pregnant individuals, except for the association with eclampsia/preeclampsia, which we will describe in a separate subchapter. Two conditions, idiopathic intracranial hypertension and cerebral venous thrombosis, are notably more prevalent in pregnant patients than in the general non-pregnant population [6,8,24].

Assessing papilledema in pregnant patients displaying symptoms indicating heightened intracranial pressure, such as pulsatile tinnitus or headaches, is vital for promptly evaluating both primary and secondary causes of ICP. Certain secondary causes, such as cerebral venous sinus thrombosis (diagnosed through neuroimaging), might be more prevalent during the third trimester and postpartum due to associated hypercoagulability. If MRI or venography does not diagnose the condition, a lumbar puncture becomes relevant to measure opening pressure and examine cerebrospinal fluid for potential secondary causes of increased ICP. Pregnant women displaying papilledema should have their blood pressure checked to rule out preeclampsia. Following the exclusion of preeclampsia, a contrast-free MRI examination (after 18 weeks) should be conducted, followed by cerebrospinal fluid analysis if not contraindicated [6,8,19,24].

Idiopathic Intracranial Hypertension (IIH) is identified as a syndrome of symptoms and indications stemming from heightened intracranial pressure, lacking observable causative lesions on magnetic resonance imaging (MRI) or computed tomography. Studies indicate a frequency of 19.3 per 100,000 in obese women of childbearing age. IIH may occasionally occur during pregnancy. Both pregnancy and exogenous estrogen are believed to either cause or exacerbate IIH. It can manifest in any trimester, and terminating the pregnancy does not necessarily halt its progression, nor does subsequent pregnancy increase the risk of recurrence. Common symptoms include headaches and vision disturbances, such as transient visual obscuration, visual field loss, and reduced central visual acuity. Due to the thrombotic risk associated with pregnancy, imaging is urgently required to exclude cerebral venous sinus thrombosis, which will be expanded on below [18,19,25].

In a study involving nine pregnant obese women, Koontz et al. reported that four experienced intensified headaches, one of whom simultaneously had increased visual acuity loss. Another case study reported that IIH symptoms, including headaches and visual changes, were exacerbated in nine pregnancies. Fortunately, many studies have reported rapid symptom improvements immediately after childbirth [25,26,27]. The therapeutic approach is similar to non-pregnant patients. There are several medications that can be administered only after 20 weeks of gestation, such as acetazolamide (a carbonic anhydrase inhibitor) used for lowering intracranial pressure, which serves as the first-line treatment. Weight reduction is crucial, and pregnant women should avoid gaining more than 20 kilograms. Pregnant women require monitoring by a nutrition specialist to track progress and establish a suitable diet. Other medications have adverse effects during pregnancy. Steroids carry unwanted side effects like weight gain, hyperglycemia, and fetal developmental disturbances; hence, they are only indicated in acute situations to treat significant visual decline. Should surgical intervention be required to prevent vision loss during pregnancy, it might entail multiple lumbar punctures or procedures for cerebrospinal fluid shunting [19,24,25,26,27]. The prognosis for individuals developing IIH during pregnancy is excellent as long as there is clinical and medical monitoring and timely surgical interventions to prevent visual impairment [24].

#### 3.2.2. Cerebral Venous Sinus Thrombosis (CVST)

Some of the venous sinuses include the superior sagittal sinus, cavernous sinus, transverse sinuses, lateral sinuses, and sigmoid sinuses. The most commonly thrombosed structures are the sagittal and transverse sinuses. They drain into the internal jugular veins, so any thrombosis at this level causes papilledema. Cerebral venous thrombosis has been linked to a wide range of predisposing factors, including pregnancy and the postpartum period. The incidence of this condition is low, but its association with pregnancy is quite significant. Several studies have been conducted, one involving 113 cases of aseptic CVST, with 67 cases happening during pregnancy or after childbirth. CVST is much more common postpartum, especially in the first two weeks after birth. Headache is the most frequent symptom, occurring in 75% of cases; when severe, it may be associated with seizures and altered consciousness. For other less severely affected patients, clinical characteristics are limited to headache and papilledema, thus mimicking the clinical picture of IIH. Therefore, ophthalmological evaluation is crucial. Brain MRI examination is also very important. Treatment targets intracranial hypertension, and it is important to differentiate the causes of venous thrombosis as anticoagulant therapy is necessary. In a separate subchapter, we will refer to medications that can be given during pregnancy according to the classification for the safety of medications used in pregnancy [24,25,26,27].

#### 3.2.3. Optic Neuropathy and Pregnancy

##### NAION and Pregnancy

Non-arteritic anterior ischemic optic neuropathy (NAION) stands as the primary reason for optic nerve swelling and neuropathy in adults aged over 50 years. Mainly associated with systemic conditions and predisposing ocular conditions, it is rarely reported during pregnancy. Santiago and colleagues reported a case of a 44-year-old healthy woman who underwent in vitro fertilization treatment to become pregnant. Eighteen days following a cesarean section without additional incidents, she encountered a sudden, painless loss of vision in her right eye (RE), which was notably denser in the inferior region. This was accompanied by optic disc swelling and a relative afferent pupillary defect. Imaging evaluation (brain MRI) and laboratory tests did not reveal a cause, and despite treatment with anti-inflammatory medication, there was no significant improvement even after 1 year of follow-up. The hemodynamic and hormonal shifts towards the end of pregnancy and an uncomplicated cesarean section might prompt an occurrence of NAION in a young, healthy woman [28]. Another case reported by Zafer Onaran and colleagues described bilateral NAION in a young patient following a massive spontaneous hemorrhage associated with an abortion, who presented sudden, pain-free loss of vision in the left eye. Acute severe hemorrhage is a key risk factor for NAION in healthy young individuals. The diagnosis was made based on clinical manifestations and MRI examination, and after treatment with intravenous methylprednisolone (1000 mg/day) for three days, there was an improvement in eyesight clarity, visual field, and resolution of papillary edema [29]. Another case described by Prashanthi Giridhar involved a 35-year-old woman who experienced a sudden loss of vision in the right eye, which was detected roughly 8 days after delivering her full-term baby vaginally. The patient had a history of preeclampsia two weeks before delivery, with high blood pressure and proteinuria. Preeclampsia can begin at 20 weeks of pregnancy with high blood pressure; ocularly, it can cause spasms and occlusion of retinal vessels, choroidal infarction, choroidal effusions resulting in serous retinal detachment, and specific edema and hemorrhages occurring in the occipital area. Generally, ocular changes are bilateral, but in this case, they were unilateral with an altitudinal field defect, a typical aspect of NAION [30].

##### Neuritis and Pregnancy

As a condition characterized by the inflammation and demyelination of the optic nerve, leading to sudden, often unilateral vision loss, optic neuritis (ON) is strongly interconnected with multiple sclerosis (MS). Atypical optic neuritis refers to instances where infectious and inflammatory causes, distinct from MS or autoimmune factors, result in similar optic nerve inflammation. About two-thirds of ON cases occur in women aged 20 to 40 years. MS, an autoimmune disorder causing nerve cell demyelination, carries an increased risk of reactivation during pregnancy. Throughout pregnancy, MS activity tends to decrease both clinically and in MRI scans, particularly in the final trimester. ON can manifest independently or serve as an early indicator of systemic demyelinating conditions like MS and neuromyelitis optica (NMO) [31].

MRI imaging and cerebrospinal fluid analysis (CSF) are conducted to rule out MS in cases of atypical optic neuritis. Oligodendrocyte myelin glycoprotein antibodies (MOG-Ab) serve as biomarkers for demyelination, significantly enhancing our comprehension of ON. Pregnancy induces complex immunological and hormonal changes crucial for fetal development, especially in autoimmune conditions like MS and NMO. Symptoms are more frequent postpartum and during the first trimester of pregnancy [9,32,33].

Wenhao Bai et al. examined the clinical subtypes and prognosis of women experiencing pregnancy-related ON at a neuro-ophthalmology unit in a Chinese hospital over nearly 5 years. Among ON instances, 11 (16.4%) arose during pregnancy, while 56 (83.6%) occurred within the initial year postpartum (PP1) or following abortion, with 33 (49.3%) happening in the first trimester. In total, 14 (25.9%) patients with pre-pregnancy ON encountered a higher relapse rate in the first year postpartum compared to the year preceding pregnancy. In two cases of AQP4-ON, patients underwent premature births and had babies with low birth weights. There were no instances of congenital defects or stillbirths reported [9].

Neuromyelitis optica (NMO) and pregnancy: Neuromyelitis optica (NMO) represents a rare demyelinating disorder within the central nervous system, causing optic neuritis and transverse myelitis. Episodes can emerge during the final trimester of pregnancy. Aquaporin-4 (AQP4)-IgG, an autoantibody thought to contribute significantly to NMO’s development by instigating astrocyte injury through complement activation, leading to myelin damage, is primarily implicated. This condition is characterized by optic neuritis and transverse myelitis. Although there might be some reduction in NMO activity during pregnancy, it appears notably less compared to that observed in MS. Studies indicate the transfer of AQP4-IgG from the mother to the fetus; however, this transmission does not appear to cause illness in the child. Similar to MS cases, there is a heightened occurrence of relapses after childbirth in NMO patients. Additionally, individuals with NMO have elevated rates of miscarriage and preeclampsia. Research demonstrates that the placenta serves as the site for maternal AQP4-IgG binding, complement deposition, and placental necrosis. In their analysis of 60 AQP4-IgG-positive individuals, Nour et al. identified a heightened miscarriage rate (43%) following the onset of the NMO spectrum disorder compared to it before. (7%). In conclusion, they suggest that pregnancy after the onset of NMOSD (neuromyelitis optica spectrum disorder) is an independent risk factor for miscarriage, and pregnancies conceived during high disease activity periods may have an increased risk of miscarriage. Women developing NMOSD and who have other autoimmune disorders have a higher chance of preeclampsia, regardless of the disease’s onset time [1,32]. Fatih Aslan describes the case of a 30-year-old woman who previously experienced NMO and underwent periodic ophthalmic evaluations via OCT to detect pregnancy’s effects on the disease. No ophthalmic changes were noted during pregnancy or postpartum. A cesarean section was preferred due to the medical history at 39 weeks [34]. Although the association between pregnancy and NMO is extremely rare, the limited available data indicate an effect on exacerbating the condition.

#### 3.2.4. Cranial Neuropathies and Pregnancy

Regarding cranial neuropathies, the most frequent is the impairment of the VII cranial nerve (facial nerve), specifically Bell’s palsy, followed by abducens and then trochlear nerve palsy. Trigeminal or oculomotor paralysis occurs less frequently. Isolated sixth nerve or abducens nerve palsy during pregnancy is uncommon, although sporadic cases have been documented. However, it is more commonly associated with gestational hypertension or preeclampsia. Bell’s palsy incidence rises during the third trimester of pregnancy, yet its exact cause remains unclear. Traditionally, facial nerve palsies during pregnancy were linked to heightened pressure on the nerve from interstitial fluid. Present recommendations propose assessing for unconventional causes and promptly initiating corticosteroids to impede progression and enhance the prognosis in such instances. In certain cases, the trochlear nerve paralysis observed during pregnancy might indicate the deterioration or breakdown of the paralysis [1,6,9,34,35] In a case report published by Marina et al., the case of a 32-year-old primiparous patient who developed preeclampsia during the birth of twins, with the occurrence of paralysis of cranial nerves V, VI, and VII on the left side, is described. Paralysis resolved completely over 3 months after treatment with valacyclovir and systemic corticosteroids [34]. The studies describe a number of neuropathies in pregnancy, the most frequent being of the seventh nerve and then of the sixth nerve. These situations can be explained by multiple mechanisms such as hypercoagulopathy, the influence of ovarian hormones, ischemia, increased extracellular fluid content, and direct or indirect effects of spinal anesthesia. Especially for pregnant patients, early preeclamptic neuropathy diagnosis and appropriate investigations should be considered [1,12,36].

#### 3.2.5. Neuro-Ophthalmic Emergencies and Pregnancy

##### Preeclampsia and Eclampsia

Preeclampsia is a multifaceted syndrome of uncertain origin characterized by hypertension (140/90 mm Hg and higher) and proteinuria, typically occurring after the 20th week of pregnancy. It is persistent high blood pressure that develops during pregnancy or the postpartum period. It is often associated with high levels of protein in the urine or the new development of decreased blood platelets, trouble with the kidneys or liver, fluid in the lungs, or signs of brain trouble such as seizures and/or visual disturbances. Eclampsia, on the other hand, is delineated as preeclampsia accompanied by seizures. Preeclampsia is thus defined as the presence of (1) a systolic blood pressure (SBP) greater than or equal to 140 mm Hg or a diastolic blood pressure (DBP) greater than or equal to 90 mm Hg or greater, twice for at least 4 hours distance in a previously normotensive patient, or (2) an SBP greater than or equal to 160 mm Hg or a DBP greater than or equal to 110 mm Hg or greater. In this case, hypertension can be confirmed within minutes to facilitate timely antihypertensive therapy. Around 25% of preeclampsia cases and 50% of eclampsia cases exhibit visual system irregularities [1,19,37,38,39,40]. Eclampsia and preeclampsia can impact any part of the visual system, spanning from the retina to the occipital cortex. Approximately a quarter of severe preeclampsia patients experience visual alterations, encompassing symptoms like diplopia, scotoma, or photopsia. The presence of convulsions or coma in a patient with preeclampsia heralds eclampsia [37,38,39]. In addition to retinal changes, these patients may develop papillary edema. Optic nerve edema may stem from intracranial hypertension, systemic hypertension, or prior ischemic optic neuropathy. Cortical visual impairment might arise due to conditions like posterior reversible encephalopathy syndrome or stroke. MRI observations in severe preeclampsia and eclampsia reveal disruptions at the gray-white boundary in the parieto-occipital region and basal ganglia. Diffusion-weighted imaging might indicate heightened signals in the occipital lobes, accompanied by apparent diffusion coefficient maps aligning with vasogenic edema rather than cytotoxic edema. An exceedingly rare incident of bilateral blindness has been documented, which was attributed to infarction in the bilateral lateral geniculate body. While the primary remedy for severe preeclampsia and eclampsia is the delivery of the new-born, obstetricians have long recognized magnesium sulfate as an effective anticonvulsant, countering cerebral vasospasm, fortifying the blood-brain barrier, and reducing cerebral edema [1,12,19]. Rismondosi et al. describe the case of a 28-year-old pregnant woman, primiparous, 37 weeks, one day before the onset of headache and nausea, without general or ophthalmological history, with a BP (blood pressure) of 166/106 mmHg, mild proteinuria, edema lower limbs, brain imaging without SC without significant changes. She was admitted to magnesium sulfate + BP monitoring. The next day, a headache and a BP of 166/104 mmHg persisted, and she declared blindness in both eyes, requiring an emergency cesarean section. The evaluation of the sight reveals the acuity of light perception, some hemorrhages in the flame at the fundus of the eyes, and otherwise normal appearance fundus. Cerebral orbital MRI with SC: hyperintense lesions in both occipital lobes. The evolution is favorable with an increase in visual acuity and changes in the visual field such as left homonymous hemianopsia; BP has normal values, and after 1 month, the MRI is normal. In this respect, the cortical blindness was temporary and probably caused by vasogenic edema rather than vasospasm. Ocular complications often forecast a positive prognosis in pregnancy-related hypertension. Nevertheless, regular assessments, timely identification, and swift intervention are crucial for ensuring favorable outcomes for both the mother and the eventual fetal well-being [15,39,40,41,42].

Cortical blindness and pregnancy. An infrequent complication of preeclampsia cortical blindness may manifest in approximately 15% of expectant mothers. The precise pathophysiological mechanism remains somewhat unclear, potentially involving cerebral vasospasm, resulting in ischemic damage and cytotoxic edema, or elevated capillary permeability contributing to vasogenic edema. Cortical blindness is associated with the absence of ophthalmological lesions anterior to the lateral geniculate body, normal ocular motility, and normal pupillary reflexes [38,39,40]. We recommend that you pay attention to any pregnant woman with headaches, hypertension or proteinuria at which a lumbar puncture, orbito-cerebral MRI should be performed: opening pressure and laboratory examination [41,42].

##### Pituitary Apoplexy and Pregnancy

An exceptionally uncommon occurrence, pituitary apoplexy leads to a sudden and intense headache coupled with neuro-ophthalmic deficits during pregnancy, typically resulting from pituitary hemorrhage or infarction. Early diagnosis is crucial because it can endanger both the life of the mother and the fetus. Diagnosing pituitary apoplexy poses challenges as it involves signs and symptoms arising from the sudden expansion of the contents within the Turkish saddle. This diagnosis is critical due to its potential to induce a neuroendocrine emergency, marked by acute central hypoadrenalism, hyponatremia, arterial hypotension, and neuro-ophthalmological deficits. Hari Sedai et al. reported a case involving a 40-year-old woman in her 21st week of pregnancy, presenting with an abrupt, severe frontoparietal headache persisting for 5 days, accompanied by projectile vomiting, right eyelid drooping, bilateral vision decline, and sensory changes over 3 days. Upon examination, the patient exhibited reduced visual acuity in the right eye, right eyelid ptosis, right afferent pupillary defect, and papillary and macular edema upon fundus evaluation. The provisional diagnosis indicated imminent eclampsia with CN III paralysis, promptly managed in a specialized center. MRI findings depicted lesions suggestive of pituitary apoplexy, confirming the diagnosis in approximately 90% of cases. Recognizing these clinical entities swiftly with the aid of a multidisciplinary team can avert dire consequences. In pregnant women, care must prioritize the well-being of both the mother and fetus, aiming to optimize the physiological stability of both individuals. The therapy is an emergency one that includes electrolytes, hormonal substitution, and neurosurgical intervention when severe neuro-ophthalmological signs appear. The prognosis can be favorable when it is diagnosed early. The current data suggest that conservative and surgical treatments have limited effects on delivery and fetal welfare. Although the long-term outcomes are not extensively documented, complete restoration of pituitary function is infrequent, potentially leading to a requirement for extended hormone replacement therapy in some individuals [1,16,43,44].

#### 3.2.6. Other Neuro-Ophthalmological Complications Associated with Pregnancy

Disseminated intravascular coagulation (DIC) is a severe condition marked by widespread clotting within small vessels, leading to subsequent hemorrhage and tissue death. It can arise in complex pregnancies involving complications such as placental abruption, preeclampsia/eclampsia, difficult deliveries, amniotic fluid embolism, intrauterine infections, and fetal demise. DIC manifests as widespread clotting in small vessels, followed by bleeding and tissue damage. At the ocular level, the choroidal layer often bears the brunt, with thrombosis in the choriocapillaries disrupting the retinal pigment epithelium, resulting in serous detachment of the retina [1,6,12]. Nishal et al. describe a case of a 40-year-old nulliparous woman who, after giving birth to twins, developed pregnancy-induced hypertension. Platelet count reduction and elevated fibrinogen levels, along with increased fibrin degradation product D-dimer (1.6 mg/mL, normal < 0.4 mm/mL), suggested the likelihood of disseminated intravascular coagulation. Notably, this condition spontaneously resolved without necessitating treatment [44]. There are no data in the specialized literature about the obstetric and fetal results in the evolution of future pregnancies.

HELLP syndrome: Marked by hemolysis, HELLP syndrome elevated liver enzymes and reduced platelet counts; it typically manifests in individuals with preeclampsia and often coincides with DIC. Among these patients, instances of serous retinal detachment, vitreous hemorrhage, central retinal vein occlusion, and Purtscher-like retinopathy have been documented. Michael W. Stewart et al. describe the case of a 25-year-old primiparous woman, during 38 weeks of pregnancy, who presented with bilateral vision loss. The patient underwent immediate hospitalization, comprehensive assessment, emergency medical intervention, and a cesarean section. Ophthalmoscopic examination revealed bilateral Purtscher-like retinopathy. Laboratory tests indicated elevated liver enzymes, thrombocytopenia, and signs of intravascular coagulation, aligning with HELLP syndrome. Although a healthy baby was delivered successfully, the patient experienced permanent vision loss [1,45].

Prolactinoma is the most common type of pituitary adenoma and a frequent cause of associated infertility, usually with amenorrhea or oligomenorrhea. It is known that the pituitary gland doubles its volume during pregnancy and returns to normal 6 months postpartum. The increase in the concentration of estrogens leads to a diffused hyperplasia of the pituitary gland [46,47]. Prolactinomas have an additional increase in volume and increased risk of compression on the optic chiasm (low risk for microadenomas and high risk for macroadenomas). CV is recommended every trimester for microadenomas and 1–2 months for macroadenomas [1,48]. The mechanisms for the appearance of neuro-ophthalmic symptoms are the increased mass effect determined by the physiological hyperestrogenism of pregnancy, which causes hypertrophy and hyperplasia of normal and pathological lactotrope cells. Glazer presents the case of a 19-year-old pregnant woman with APP prolactinoma, secondary amenorrhea, without ophthalmological manifestations, and serum PRL (prolactin) 520 ng/mL. After 12 months of treatment with carbegoline 1.5 mg/week, she presented with regular periodics and PRL is 19 ng/mL, and MRI appearance with tumor regression. It returned in the third month of pregnancy and carbegoline was stopped. Over a month, she complained of severe headaches without eye damage and PRL 110 ng/mL; the MRI showed an increase in tumor size. Bromocriptine was started, the headache was reduced, and the PRL level was 46 ng/mL. The evolution was favorable and the birth was due by cesarean section. Pregnancy is associated with changes in the hypothalamic–pituitary axis and targets the glands and tissues. But thanks to the current progress in medical and surgical technology, pregnancy is also possible in women with pituitary adenomas [49].

Meningiomas: Gestational meningioma occurs at a rate of 5.6 cases per 100,000 pregnant individuals. Although they know a slowly progressive evolution, during pregnancy they can be aggressive. Increased estrogen, progesterone, and prolactin levels can stimulate the rapid growth and vascularization of pre-existing tumor formations. In the second and third trimesters of pregnancy, the most significant tumor progression occurs. Postpartum, a decrease in the tumor volume can be observed, which can reappear in the following pregnancies. The existence of meningiomas during pregnancy can result in complex clinical scenarios. Their location and size may or may not manifest themselves clinically, with secondary HTIC or compression of neural structures. Observation is recommended until birth. There is only an intervention in malignant meningiomas, in the case of hydrocephalus, or when life is endangered [50,51,52,53,54]. Lee describes the case of a 26-year-old primiparous pregnant woman, 8 months pregnant, who complained of decreased AVOS, a headache that increased in intensity during pregnancy, and persistent nausea and vomiting with onset in the 2nd month: LE blurred vision that progressed. The vision at OS was up to the movements of the hand, with a related pupillary deficit on the left. Fundus examination revealed partial optic nerve atrophy in the right eye and total optic nerve atrophy in the left eye. As the therapeutic procedure, a cesarean section and a transcranial resection of an encapsulated suprasellar tumor formation were performed. The anatomical-pathological examination provided the diagnosis of meningioma. The postoperative evolution was slightly favorable with the improvement of visual acuity, with the neuro-ophthalmological evaluation at 1 year being stationary. Effective collaboration among the ophthalmologist, neurosurgeon, obstetrician, and neonatologist is crucial for achieving the best possible outcome. While surgical removal remains the preferred treatment, hormone therapy could be beneficial for managing meningiomas that cannot be completely resected or partially resectable cases [1,50,51,52,53,54].

Arterial microaneurysm: Cerebral arteriovenous malformation (CAVM) is a rare congenital condition that poses life-threatening risks of rupture during pregnancy. Prolonged, persistent nausea and vomiting beyond the early second trimester often manifest as common symptoms of CAVM during pregnancy. Deciding the optimal timing and method of delivery after managing CAVM during pregnancy remains unclear. A case involving a 25-year-old primipara illustrates this challenge: at 17 weeks gestation, she experienced persistent nausea and vomiting for a month, leading to lethargy and dizziness. Medical tests revealed normal results except for urinary ketones. Four days later, she developed muscle weakness in all limbs, preceded by a severe headache and papillary edema. Neurological exams indicated intact cranial nerves, but an MRI showed subacute CAVM bleeding in the left parietal region. After careful assessment, surgical intervention—craniotomy, hematoma evacuation, and CAVM excision at 24 weeks of gestation—was deemed beneficial despite maternal and fetal risks. Treatment with Tegretol and physical therapy followed. At 38 weeks gestation, she delivered a healthy 2.8 kg boy via elective cesarean section. Both mother and child were discharged 10 days after birth. Determining the delivery mode and timing relies on the multidisciplinary team’s decision during pregnancy. Conservative management is suitable for unruptured AVMs without increased bleeding risks. MRI and angiography play crucial roles in evaluating vascular abnormalities and identifying feeding vessels for the AVM during pregnancy. Immediate symptom recognition and imaging are vital. Successful outcomes demand a collaborative approach involving neurosurgeons, obstetricians, and anesthesiologists to ensure maternal and fetal welfare [1,55].

#### 3.2.7. Neuro-Ophthalmological Procedures in Pregnancy

Typically, the assessment would proceed similarly as if the woman were not expecting. Our approach assumes that by correctly diagnosing and treating the mother, she will responsibly attend to the well-being of the fetus. We employ identical diagnostic tools as we would in any neuro-ophthalmology assessment to reach a diagnosis, which we describe in Table 2 [1,6,7,56]. Given the absence of standard contraindications, an MRI is generally deemed safe and the preferred imaging method during pregnancy. However, all FDA-approved gadolinium chelates are categorized as FDA Class C. As a result, gadolinium is reserved for urgent scenarios where there is a critical need to assess potentially life-threatening conditions for either the fetus or the mother. Even in these cases, it is typically avoided until after the first trimester. Computed tomography is conducted with proper abdominal shielding to minimize radiation exposure, especially in early pregnancy. Iodinated contrast dye is categorized as Class B (animal studies suggest some risk, but there is no definitive evidence of risk in humans). While fluorescein and indocyanine green used in retinal angiography fall under Class C, fluorescein crosses the placenta and enters breast milk, whereas indocyanine green has no impact [1,6,7,56].

#### 3.2.8. General Neuro-Ophthalmic Medication and Pregnancy

No study has prospectively evaluated the safety of drugs in pregnancy. A significant portion of our knowledge stems from animal studies and reports on toxicity. The primary categorization of medications during pregnancy originates from the FDA classification system and is detailed in Table 3 [1,6,56].

In Table 4, we detail the drugs useful for neuro-ophthalmic pathology, during pregnancy when they can be administered, and the duration of administration. The input of obstetricians, ophthalmologists, and family physicians is crucial to ensure the safe use of drugs during pregnancy. Close monitoring of maternal and fetal conditions during treatment is imperative. It is particularly important to avoid these medications during the initial trimester of pregnancy due to a higher risk of drug-induced fetal abnormalities during this period compared to others.

Other drugs used for neuro-ophthalmic diseases: For the analgesics available to treat headaches, meperidine and acetaminophen with codeine are recommended for short-term use, while propranolol and topiramate can be used prophylactically for severe intractable headaches.

The author emphasizes the importance of comprehending medication risks to effectively manage neuro-ophthalmic complications during pregnancy. Aspirin (FDA class C) might be advised in certain cases, while prophylactic anticoagulation with heparin or heparinoids (FDA class B) might be indicated in others. Generally, Warfarin (FDA class X) is avoided due to associated fetal malformations from concurrent fetal anticoagulation. Though no teratogenic effects are reported from using dilating drops or topical anesthetics during pregnancy, caution is urged with systemic use of atropine, homatropine, or phenylephrine in early pregnancy, as they have links to fetal malformations. Their use, even for examination purposes, is not recommended during the initial three months of pregnancy [1,16,57]. Case reports detail the use of anti-VEGF intravitreal injections during pregnancy [35,36]. In one case, photodynamic therapy (PDT) occurred within the first two weeks of pregnancy, followed by 1.25 mg intravitreal bevacizumab in the third month, resulting in a completed pregnancy without complications. Another case received intravitreal bevacizumab in the third trimester without issues [57]. Verteporfin is classified ascategory C. For pregnant patients requiring unavoidable ocular surgery, lidocaine and citanest, both class B drugs, might be the safest anesthetics. However, bupivacaine, a category C drug, is not recommended due to potential fetal bradycardia risk. Similarly, proparacaine hydrochloride, used as a topical anesthetic, falls under this category [57].

## 4. Conclusions

Pre-existing ophthalmological and neuro-ophthalmological conditions can be worsened or ameliorated due to a variety of physiological changes induced during pregnancy. Visual disturbances are highly common in pregnant women.

Uncommon yet severe complications might involve visual symptoms, prompting initial consultation with an ophthalmologist for pregnant patients. The evaluation and treatment of a pregnant woman with neuro-ophthalmic manifestations are challenging. The obstetrician is the one who follows and has a close medical relationship with the pregnant woman; therefore, he/she can be the first to be informed about the general condition of the pregnant woman or can request an ophthalmological consultation adapted to each individual case.

Early diagnosis of neuro-ophthalmic emergencies like pituitary apoplexy and preeclampsia/eclampsia is crucial as it can jeopardize both the mother’s and the fetus’s lives. We recommend paying attention to any pregnant woman experiencing headaches, elevated blood pressure, and proteinuria, thereby requiring an urgent orbital-cerebral MRI.

The scientific evidence concerning the risk of administering ophthalmic drugs to pregnant women is generally insufficient. There is a notable absence of meta-analyses and controlled studies in this specific area. Treating most of these conditions aligns with non-pregnant patient protocols, yet considering potential teratogenic effects, treatment profiles, and therapy administration requires careful consideration.

The treatment of neuro-ophthalmic complications of pregnancy requires an understanding of the risks of drugs. Taking optimal care of the mother will usually lead to the best care for the baby.

## Figures and Tables

**Table 1:** Effects of pregnancy on pre-existing ophthalmic conditions.

Conditions	The Effect Caused by Pregnancy
Glaucoma	Variable outcomes despite reduced IOP during pregnancy [6,19]: - 57% reported stable IOP and normal visual fields. - 18% reported visual field loss despite stable IOP
Uveitis	The decline in the occurrence rate of uveitis [19,20]
Diabetic retinopathy	The risk of exacerbating non-proliferative diabetic retinopathy decreases by 5% in the third trimester towards proliferative retinopathy. The advancement of diabetic retinopathy is linked to high blood pressure and diabetic kidney disease [6,19,21]
Obstruction of retinal blood vessels	Could arise as a consequence of disseminated intravascular coagulation caused by HELLP syndrome, V Leiden factor mutation, and thrombophilia. Both hereditary and acquired thrombophilia not only heighten the risk of maternal thrombosis but also increase the likelihood of miscarriage or eclampsia [3,6,22].

**Table 2:** Diagnostic procedures in neuro-ophthalmology. IRM (intracranian magnetic resonance), CT (computer tomography), risk to the mother, and risk to the fetus [1,6,7,56].

Test	Risk to Mother	Risk to Fetus	Contraindication	Notes
IRM	None	None known	The presence of metals in the body	Gadolinium falls under FDA class C.
CT	None	Minimal	Dye allergy	Provide shielding for the abdomen; iodinated contrast dye falls under class B classification.
Angiograma	None	Minimal	Dye allergy	Provide shielding for the abdomen
Spinal tap	None	None	Incipient herniation in the mother	-
Fluorescein angiogram falls under FDA Class C categorization.	None	None known	Allergy	It crosses the placenta barrier and can also pass into breast milk.
Indocyanine green angiography is categorized as FDA Class C.	None	None known	None known	It does not cross the placenta and is safe for use during pregnancy.
Tropicamide, the dilating drops, is classified as FDA Class C.	None	None known	Closed-angle glaucoma	Close off the puncta while administering.

IRM (intracranian magnetic resonance), CT (computer tomography), risk to the mother, and risk to the fetus. Food and Drug Administration (FDA).

**Table 3:** FDA classification of medications that can be used during pregnancy; in alignment with the Food and Drug Administration (FDA). https://www.fda.gov/consumers/womens-health-topics/medicine-and-pregnancy (free access).

FDA CLASS	Risks
A	Studies under control conditions indicate there is no identified risk. It is the safest category.
B	Animal studies suggest a potential risk, yet no conclusive evidence of risk has been observed in humans. Can be used if necessary.
C	The risk factor is uncertain but not entirely ruled out (typical for most drugs). Nevertheless, the potential benefits might outweigh the potential risks, justifying the drug’s use in pregnant women.
D	Research in animals or humans indicates a potential risk; yet in certain cases, the benefit might surpass this risk. The medication could be cautiously employed if not using it poses higher risks for both the mother and the fetus.
X	Research in animals or humans demonstrates significant fetal risk that clearly outweighs any potential benefit. Avoid prescribing drugs categorized as “X”. Use is not recommended.

Food and Drug Administration (FDA).

**Table 4:** Medications used in neuro-ophthalmic disorders, drug type, duration of pregnancy, duration of administration, and references. NMO (optic nerve neuromyelitis), IIH (idiopathic intracranial hypertension).

Neuro-Ophthalmic Pathology	Drug Type	Duration of Pregnancy	Effects	Duration of Administration	References
Pituitary adenoma	Bromocriptine, dopamine agonist	start it after the first trimester	It passes through the placenta and should be employed only when absolutely needed. these medications impair lactation.	Only if is necessary	[1,16]
Multiple sclerosis	interferon-b [FDA] class C, glatiramer acetate (FDA class B,	Frequently recommended during pregnancy and for a period prior to conception.	Are generally considered safe for use throughout pregnancy, having minimal impact on the fetus.	Resumed right after giving birth to prevent relapse. It is advised to avoid breastfeeding during this period.	[1,16]
NMO	Corticosteroids and plasma exchange can be safely used. Chronic immunosuppression	It is recommended to use them at the lowest effective doses. Depending on the specific agent, it might be beneficial to sustain immunosuppression throughout pregnancy.	Teratogenic effects: topical steroids have no known teratogenic effects	Use these medications at the minimum effective doses. Discontinue them before attempting conception: mycophenolate mofetil should be stopped 6 weeks before, whereas methotrexate and cyclophosphamide should be halted 3 months prior.	[1,16]
IIH	Acetazolamide FDA class C Topiramate is FDA class D Furosemide is FDA class C	Is avoided during pregnancy; should not be used in pregnancy	In animal studies, administering high doses of acetazolamide has demonstrated the potential to cause birth defects, including (forelimb anomalies observed in rats, mice, and hamsters). Before 13 weeks, risk of spontaneous abortion is correlated with the development of cleft lip and cleft palate if taken in the first trimester.	After 13 weeks of pregnancy	[1,16,57,58,59]
CEREBRAL VENOUS SINUS THROMBOSIS	Unfractionated heparin Warfarin, it is FDA class X,	Lowers the likelihood of mortality in pregnant women diagnosed with CVST; should not be used in pregnancy	Devoid of substantial maternal or fetal side effects, it is usually avoided due to potential fetal malformations and abnormalities resulting from simultaneous fetal anticoagulation.	The use of low-molecular-weight heparin as a preventive measure during pregnancy and the period surrounding childbirth should be considered.	[1,16,58,59]
PREECLAMPSIA AND ECLAMPSIA	Intravenous magnesium sulfate, as an anticonvulsant	Remains the mainstay of therapy	Can lead to neuro-ophthalmic signs and symptoms, relaxing accommodation, reducing convergence, and inducing ptosis.		[1,16,58,59]

NMO (optic nerve neuromyelitis), IIH (idiopathic intracranial hypertension), CVST (cerebral venous sinus thrombosis).

## Data Availability

The datasets used and analyzed during the current study are available from the corresponding author upon reasonable request.
